# Versatile Structures of α-Synuclein

**DOI:** 10.3389/fnmol.2016.00048

**Published:** 2016-06-20

**Authors:** Chuchu Wang, Chunyu Zhao, Dan Li, Zhiqi Tian, Ying Lai, Jiajie Diao, Cong Liu

**Affiliations:** ^1^Interdisciplinary Research Center on Biology and Chemistry, Shanghai Institute of Organic Chemistry, Chinese Academy of SciencesShanghai, China; ^2^Department of Cancer Biology, College of Medicine, University of CincinnatiCincinnati, OH, USA; ^3^Center for Mitochondrial Biology and Medicine, Key Laboratory of Biomedical Information Engineering of Ministry of the Education School of Life Science, Xi’an Jiaotong UniversityXi’an, China; ^4^Department of Molecular and Cellular Physiology, Stanford UniversityStanford, CA, USA

**Keywords:** α-synuclein, Parkinson’s disease, amyloid, atomic structure, vesicle trafficking, protein aggregation

## Abstract

α-Synuclein (α-syn) is an intrinsically disordered protein abundantly distributed in presynaptic terminals. Aggregation of α-syn into Lewy bodies (LB) is a molecular hallmark of Parkinson’s disease (PD). α-Syn features an extreme conformational diversity, which adapts to different conditions and fulfills versatile functions. However, the molecular mechanism of α-syn transformation and the relation between different structural species and their functional and pathogenic roles in neuronal activities and PD remain unknown. In this mini-review, we summarize the recent discoveries of α-syn structures in the membrane-bound state, in cytosol, and in the amyloid state under physiological and pathological conditions. From the current knowledge on different structural species of α-syn, we intend to find a clue about its function and toxicity in normal neurons and under disease conditions, which could shed light on the PD pathogenesis.

Abnormal aggregation of α-synuclein (α-syn) is closely associated with Parkinson’s disease (PD). The aggregated α-syn was found dominantly in Lewy bodies (LB) as the hallmark of PD (Spillantini et al., 1997) and other syncleinopathies such as dementia with LB, and multiple system atrophy. α-Syn also co-aggregates with amyloid β (Aβ) in the brain tissue of patients diagnosed as Alzheimer’s disease (AD; Uéda et al., 1993). Various point mutations of gene *SNCA* (encoding α-syn), and the duplication and triplication of α-syn locus all lead to dominant familial PD (Polymeropoulos et al., 1997; Krüger et al., 1998; Singleton et al., 2003; Chartier-Harlin et al., 2004; Zarranz et al., 2004; Mizuno et al., 2012; Lesage et al., 2013; Proukakis et al., 2013; Pasanen et al., 2014; Ferese et al., 2015). Despite its crucial role in pathogenesis, α-syn is believed to perform biological functions under physiological conditions because of its extremely high abundance in primary neurons, representing 1% of the total protein (Mizuno et al., 2012). It mainly gathers in presynaptic terminals and associates with reserve pool of synaptic vesicles (Maroteaux et al., 1988; Larsen et al., 2006; Nemani et al., 2010). α-Syn is capable of clustering synaptic vesicles and facilitating the assembly of soluble N-ethylmaleimide-sensitive factor attachment protein receptor (SNARE) complex (Burré et al., 2010). Corresponding to its diverse roles under both physiological and pathological conditions, various forms of α-syn, such as monomer, oligomer, and fibril, were found in cytosol, on membrane, and in the amyloid aggregation states (Iwai et al., 1995; Burré et al., 2014; Theillet et al., 2016). Therefore, to reveal the structural variations of α-syn is of great importance to understand its physiological function and pathogenic property.

α-Syn is an intrinsically disordered protein. It consists of 140 amino acids and is divided into three regions (Figure 1A). Each region has distinct physiochemical property corresponding to the amino acid composition. The N-terminal region (residues 1–60) is capable of forming an amphipathic helix which is a typical conformation for membrane recognition and association (Segrest et al., 1990; Jao et al., 2008). Several mutations found in familial PD, such as A53T (Polymeropoulos et al., 1997), E46K (Zarranz et al., 2004), and A30P (Krüger et al., 1998), are located in this region, indicating the importance of membrane binding for the function of α-syn. The central region containing residues 61–95 is well known as the non-amyloid β component (NAC) firstly identified in AD senile plaques (Uéda et al., 1993). This region features a high propensity to form a β-rich conformation and is highly aggregation-prone (Uversky et al., 2001). Different types of post-translational modifications within this region show distinct effects on modulating α-syn aggregation. For example, glycosylation at T72 and phosphorylation at S87 have been reported to block α-syn aggregation (Paleologou et al., 2014; Marotta et al., 2015). The C-terminal region with residues 96–140 is rich of proline and negatively charged residues including 10 glutamates and five aspartates, which is a common characteristic found in intrinsically disordered proteins to maintaining solubility. On the structural front, several important questions remain unclear. How do the three regions independently and cooperatively accomplish the formation and conversion of α-syn structures in different states? What are the common structural features of α-syn in different states? How to correlate the biological function and cellular toxicity of α-syn with the different structures or structural transformation? Regarding these important fundamental questions, we summarize the current knowledge of α-syn structures in the states of membrane binding, cytosol, and amyloid aggregation. We also discuss the relationship between the different structural states and their physiological function and pathological toxicity.

**Figure 1:**
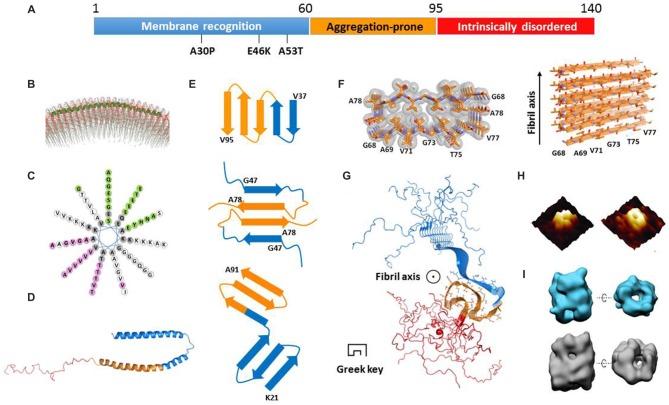
**Structural models of α-synuclein (α-syn) in different states. (A)** The primary sequence of α-syn with three regions highlighted in blue, orange and red, respectively. The three familial Parkinson’s disease (PD)-related mutations are denoted. **(B)** The structural model of an α-syn monomer on unilamellar vesicles (from Jao et al., 2008 reprinted with permission). The continuing helix (residues 9–89) is colored in green. **(C)** The helical wheel model of α-syn membrane-binding region from residue 9 to 90. **(D)** The structure of a micelle-bound α-syn monomer (Protein Data Bank ID: 1XQ8). The three regions are color-coded according to **(A)**. **(E)** The geometry and arrangement of a single layer of fibril core from three α-syn fibrillar models in cartoon form (Vilar et al., 2008; Dearborn et al., 2015; Rodriguez et al., 2015) viewed perpendicular to the fibril axis. The top and bottom ones both contain single α-syn monomer per layer of protofilament. The middle one contains two α-syn monomers paired via steric-zipper-like interaction. The different regions are color-coded according to **(A)**. **(F)** The atomic structure of α-syn non-amyloid β component (NAC) fibril core obtained from electron diffraction (Protein Data Bank ID: 4RIL). **(G)** The structural model of an α-syn fibril determined by solid-state nuclear magnetic resonance (NMR; Protein Data Bank ID: 2N0A). The three regions are color-coded according to **(A)**. The side chains of the fibril core (residues 46–54 and 63–96) are shown in stick representation. **(H)** The architecture of an α-syn oligomer formed in the presence of supported lipid bilayers probed by atomic force microscopy (AFM; from Quist et al., 2005 reprinted with permission). **(I)** The structural model of two types of soluble α-syn oligomers (upper for 10S and bottom for 15S oligomer subgroups) measured by cryoEM (from Chen et al., 2015 reprinted with permission).

## The Structure of Membrane-Bound α-Syn

α-Syn is capable of binding to membrane upon the structural transition from random coil to α-helix (Davidson et al., 1998). The N-terminal region of α-syn contains seven copies of a tandem 11-residue pseudorepeat, which is a characteristic of lipid-binding domains found in apolipoproteins (George et al., 1995). By using electron paramagnetic resonance (EPR) spectroscopy and nuclear magnetic resonance (NMR) spectroscopy, α-syn was found to adopt a continuous helix from residue 9–89 on the surface of unilamellar vesicles (Figure 1B; Jao et al., 2008; Bodner et al., 2009). As shown in the helical wheel (Figure 1C), the helix features four interfaces with distinct properties. The interface in pink is mainly composed of hydrophobic residues and is buried inside the membrane. The green interface containing several Glu residues is exposed to the solvent. Meanwhile, the two white colored strips on the two sides of the helix feature 11 positively charged Lys residues which correspond to the electrostatic interaction with the head groups of lipids. Indeed, it was reported that α-syn only recognizes and binds to the membrane composed of phospholipids with negatively charged head groups such as PS and PA (Eliezer et al., 2001; Jain et al., 2013).

A recent work shows that, upon membrane recognizing and binding, the first 25 amino acids are involved in a well-defined helical structure acting as a membrane anchor to initiate docking of α-syn on the membrane (Fusco et al., 2014). Meanwhile, the rest of the helix (residues 26–97) exhibits a structural flexibility, and transforms between helix and random coil depending on the lipid composition (Fusco et al., 2014). This region is believed to determine the binding affinity of α-syn to membrane. Upon binding to a highly curved membrane surface, the continuous helix breaks into two individual helices (Rao et al., 2010). The structure of α-syn bound to sodium dodecyl sulfate (SDS) micelles was determined by using solution NMR (Ulmer et al., 2005). Unlike the continuous helical structure found in the liposome-bound state, SDS-bound α-syn forms two slightly curved α-helices (residues 3–37 and 45–92) exhibiting a hairpin structure connected by a stretched five-residue linker (Figure 1D). The circle diameters of the two curved α-helices are ~153 Å and ~82 Å, respectively, which are much smaller than that of presynaptic vesicles (~400–500 Å). No direct interactions were observed between the two helices. Molecular dynamics (MD) simulations show that, depending on the curvature and property of the membrane surface, α-syn senses and adapts to membrane by adjusting the circle diameters and the relative positions of the two helices (Drescher et al., 2008; Ferreon et al., 2009; Lokappa and Ulmer, 2011; Robotta et al., 2011). In the membrane-bound state, the C-terminal region is highly flexible with no association to the membrane. In addition, a recent study indicated that α-syn forms a multimer on the membrane by using fluorescence resonance energy transfer (FRET) and chemical cross-linking (Burré et al., 2014). However, the atomic structural information of this multimer is absent.

## The Function of α-Syn in Synaptic Transmission

Studies have suggested that α-syn functions in various aspects of synaptic transmission. Burré et al. (2010) have found that α-syn can facilitate neuronal SNARE complex assembly, which is fulfilled by the direct interaction between the highly flexible and negatively charged C-terminal region of α-syn and the N-terminus of synaptobrevin-2/VAMP2 (vesicle associated membrane protein 2; Burré et al., 2010). Synaptobrevin-2/VAMP2 is a key component of SNARE complex residing on the synaptic vesicles. Membrane anchoring of α-syn via its N-terminal region is also essential for the process. The same group has also hypothesized that, in compensation for cysteine string protein α (CSPα), α-syn can act as a neuroprotective chaperone to maintaining the SNARE function (Chandra et al., 2005).

Complementary to the *in vivo* studies, *in vitro* assays involving protein-reconstituted vesicles were performed to investigate the function of α-syn in synaptic transmission (Brunger et al., 2015). Diao et al. (2013) showed that α-syn significantly promotes the clustering of protein-reconstituted liposomes—minimal mimics of synaptic vesicles, while with little effect on Ca^2+^-triggered fusion in a single vesicle-vesicle system with reconstituted neuronal SNAREs, synaptotagmin-1, and complexin-1. The function of vesicle-clustering was observed for both recombinant as well as native α-syn purified from mouse brains, and was confirmed by *in vivo* studies (Wang et al., 2014). The clustering of synaptic vesicles by α-syn could promote the neuronal trans-SNARE complex formation and provide a “buffer” of synaptic vesicles for synaptic transmission.

α-Syn was also found to reduce ensemble lipid mixing (Dewitt and Rhoades, 2013). Through advanced single vesicle technique, others found that α-syn reduces the ensemble lipid mixing by blocking vesicle association (Choi et al., 2013; Lai et al., 2014). α-Syn was also shown to jam the synaptic vesicle trafficking on the “priming” step (Larsen et al., 2006). Overexpression of α-syn in chromaffin cells resulted in the accumulation of docked vesicles without being secreted (Larsen et al., 2006). Another study showed that α-syn impairs the re-clustering of synaptic vesicles on the vesicle recycling step after endocytosis in the rodent hippocampus (Nemani et al., 2010). Therefore, α-syn can play multiple roles throughout the process of synaptic vesicle trafficking, including vesicle clustering, priming, fusion (assembly of SNARE complex), and recycling (disassembly of SNARE complex; Lashuel et al., 2013).

These functions of α-syn are all associated with membrane binding, while the structural information of membrane-bound α-syn described in the early section is too limited to explain the underling mechanism. However, the versatile functions may reflect a dynamic conformational transition of α-syn possibly combined with an association and dissociation of α-syn multimers upon interacting with membranes. Therefore, it is reasonable to deduce that α-syn is regulated by some unidentified cofactors to ensure proper structural conformations, cellular localization, and local concentration upon membrane binding and release. The failure of regulation might result in dysfunction of α-syn and amyloid aggregation.

## α-Syn in Cytosol Under Physiological Conditions

Although α-syn exhibits a strong membrane-binding capability, accumulating evidence show that α-syn dominantly exists in cytosol instead (Binolfi et al., 2012; Fauvet et al., 2012; Theillet et al., 2016). However, studies of α-syn in cytosol gained conflicting results. Recombinant α-syn purified from *E. coli* showed an unstructured monomeric species (Weinreb et al., 1996). Endogenous α-syn extracted from neurons (M17D), HeLa, brain tissues, and human red blood cells was characterized dominantly as a tetramer featuring an α-helical bundle structure that were evidenced by multiple approaches including native PAGE, cross linking, transmission electron microscopy (TEM), scanning transmission electron microscopy (STEM) and sedimentation equilibrium analytical ultracentrifugation (SE-AUC; Chen et al., 2007; Bartels et al., 2011). However, following studies by other biophysical methods (e.g., light scattering and NMR) indicated that the “tetramer” species was actually a minority compared to dominant unstructured monomers (Binolfi et al., 2012; Burré et al., 2013; Gurry et al., 2013). Recently, Theillet et al. (2016) have shown that α-syn remains as a monomer by using *in vivo* NMR (Theillet et al., 2016). They transformed mammalian neuronal and non-neuronal cells with ^15^N labeled α-syn by electroporation, and found that most regions of α-syn remain unstructured with a high flexibility. An intriguing observation is that, the intensity of NMR signals of the N-terminal 10 residues and a few residues in the central and C-terminal regions of α-syn dropped dramatically, indicating an interaction between α-syn and some unidentified molecules *in vivo* (Theillet et al., 2016). Theillet et al. (2016) tested the interactions of Ficoll, BSA, lysozyme, and liposome with α-syn *in vitro*. However, none of them can fully resemble the *in vivo* observation. The controversial results of the structural studies on the native state of α-syn in cytosol suggest a significant morphological plasticity. It is plausible that the structure of α-syn in cytosol highly depends on its binding partners such as metabolites, lipids, and chaperones. The identification of these binding partners of α-syn in cytosol would be a notable step forward to understanding the physiological function of α-syn.

## Polymorphic Structures of the Parkinson’S Associated α-Syn Amyloid Aggregation

Under certain conditions, α-syn spontaneously enters the amyloid state and self-assembles into amyloid oligomers and fibrils. Amyloid fibril of α-syn was identified as a prominent component of LB, a molecular hallmark of PD (Spillantini et al., 1997). It remains controversial whether the α-syn amyloids are the consequence or cause of PD. Therefore, structural understanding of α-syn amyloids is crucial to imply the cytotoxicity of the amyloid aggregates and to reveal the molecular mechanism of the disease. However, given the intrinsic instability and polymorphic nature of amyloid oligomers (Stroud et al., 2012; Gurry et al., 2013), and insolubility and heterogeneity of amyloid fibrils (Heise et al., 2005; Liu et al., 2012), it remains challenging to determine the atomic structures of full-length α-syn in either form.

Despite the difficulties, efforts have been made towards the atomic structures of α-syn fibrils by using different approaches. The fibril core was firstly mapped by proteinase K digestion of recombinant α-syn and α-syn extracted from synucleinopathic brains (Miake et al., 2002). The fibril core contains residues 31–109, covering the entire NAC region and part of the N- and C-terminal regions. The structural model of α-syn protofilament was calculated by the structural constraints derived from EPR (Der-Sarkissian et al., 2003; Chen et al., 2007) and solid-state NMR experiments (Heise et al., 2005; Vilar et al., 2008). This model comprises a five-layered β-sandwich, which generates five layers of parallel, in-register β-sheets as incorporated into a protofilament. Furthermore, protofilaments align with or twist around each other to form a fibril (Figure 1E, upper). This model sets up a starting point toward the structural understanding of α-syn amyloid aggregation.

Recently, micro-electron diffraction (MicroED) has been applied to the micro-sized and nano-sized crystals of amyloid-forming peptides. Atomic structures of the segments from α-syn fibril core, NACore (residues 68–78), and PreNAC (residues 47–56), were determined at 1.4 Å resolution (Rodriguez et al., 2015). The structure of NACore in the fibrillar form reveals a typical steric zipper spine structure featuring a high complimentary and dry interface formed by Thr and Ile of the two adjacent 11 mers (Figure 1F). Importantly, the NACore fibril resembles the cytotoxicity of the full-length α-syn fibril in the PC12 cells. Based on the atomic structures of NACore and PreNAC, a hypothetical model of α-syn fibril was built. In this model, each α-syn monomer forms an antiparallel β-sheet structure stabilized by a hetero-zipper-like interaction, and stacks on top of each other to form a protofilament (Figure 1E, middle). Two protofilaments are paired simultaneously by zipper-like interactions of residues V70, T72, V74, and A76 (Figure 1E, middle). The model explains the disease-related mutation A53T, which could form a tighter steric zipper interface so that stabilizes the toxic fibril structure. This is also in consistence with the highly aggregation-prone nature of A53T, which may gain higher resistance to degradation than the wildtype.

Another structural model of α-syn protofilament was proposed based on cryo-EM and dark-filed STEM studies (Dearborn et al., 2015). Each protofilament consists of two v-shaped β-serpentines formed by three antiparallel β-strands (Figure 1E, bottom). The entwined protofilaments may further assemble into mature fibrils. However, the resolution of the structural model is low (~30 Å) owing to the heterogeneity of the fibril samples. Detailed structural information is still beyond reach from this study. Most recently, a three-dimensional structural model of full-length α-syn fibril was determined by solid-state NMR and STEM, and validated by fibril diffraction (Figure 1G; Tuttle et al., 2016). The root-mean-square (RMS) deviation of the 10 lowest-energy structures of fibril core (residues 46–54 and 63–96) is 2.0 Å for all heavy atoms. The structure features a Greek-key topology which is entirely different from previous fibril structures. The fibril core is formed by parallel in-register β-sheets, and stabilized by hetero-steric zippers, salt-bridges, a glutamine ladder and hydrophobic interactions. Notably, the fibrils used in this structural study can induce aggregation of endogenous α-syn to form phosphorylated inclusions, and cause neuronal injury and death of primary hippocampal neurons. The follow-up study on structure-toxicity relationship will be valuable to elucidate the structural basis of α-syn fibril pathology.

The diverse structures of α-syn fibril support the polymorphic nature of α-syn fibrils formed under different conditions. Recently, two strains of α-syn fibrils with distinct morphologies were reported (Bousset et al., 2013). They exhibit different neurotoxicity and cell-to-cell transmission properties, and thus lead to distinct pathologies. Another study shows that α-syn is capable of cross-seeding the aggregation of tau protein, which is one of the main amyloid proteins in AD, *in vivo* (Guo et al., 2013). Notably, tau proteins cross-seeded by α-syn fibrils prepared from various protocols exhibit different properties and result in distinct histopathological and behavioral phenotypes in mice. Therefore, it would be valuable to link structural models of α-syn fibrils with their pathogenic counterparts, which may possibly imply the difference of the cellular effects, and bridge the gap between structures and neuronal toxicity.

The amyloid oligomers of α-syn were reported to cause cell death in cells and model systems such as *C. elegans* and *Drosophila* (Karpinar et al., 2009). In contrast to the fibrillar form of α-syn, the structural details of α-syn oligomers remain absent owing to their transient and labile properties. Several studies report that α-syn oligomers feature a β-rich structure with either parallel or antiparallel β-sheets. An annular channel-like architecture of oligomers formed by wild-type α-syn or A53T mutant was observed in the presence of lipid by atomic force microscopy (AFM; Figure 1H; Quist et al., 2005) and in solution by EM (Lashuel et al., 2002). It indicates that α-syn oligomers may poison cells by forming ion channels on the membrane or disrupting the membrane integrity. Through cryo-EM, another group characterized α-syn oligomer structures showing two types of annular oligomers in solution (Figure 1I; Chen et al., 2015). The cavity in these annular structures may accommodate substrates such as metals, fatty acids, and other small molecules. Interestingly, α-syn point mutations E35K and E57K, rather than A30P and E46K, prefer to form annular oligomers, are more prone to form fibrils, and exhibit potential neuronal toxicity in the rat substantia nigra (SN) region (Winner et al., 2011). Most recently, a label-free single molecular study revealed that lipids facilitate α-syn oligomerization without altering the aggregation pathway (Hu et al., 2016). These results together highlight the potential importance of annular oligomers in pathogenesis.

## Outlook

**Figure 2:**
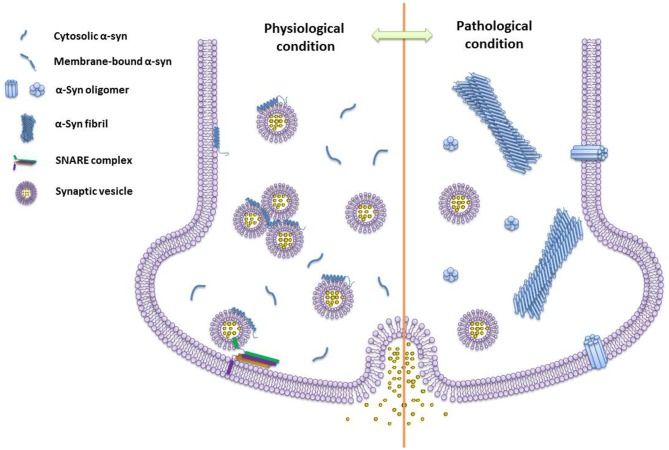
**The schematics of different species of α-syn under physiological and pathogenic conditions.** Monomeric α-syn induces vesicle clustering and chaperones soluble n-ethylmaleimide-sensitive factor attachment protein receptor (SNARE)-complex assembling via binding to the SNARE protein synaptobrevin-2/VAMP2 and membrane under the physiological condition. While, under the pathogenic condition, α-syn self-assembles into amyloid oligomers and fibrils. Different types of α-syn oligomers may impair vesicle association, and insert into the membrane by forming pore-like oligomers.

The research works summarized here show a rapid advance of our knowledge on α-syn functions and structures under physiological and pathogenic conditions (Figure 2). Meanwhile, several fundamental questions remain to be answered, such as the structural basis of α-syn conformational transition, the correlation between structural models and the cellular roles of α-syn, as well as unknown endogenous cofactors (e.g., metabolites, lipids, and chaperones) regulating the structural transformation of α-syn upon environmental changes. New technologies would be extremely helpful to address these questions. For example, integrating new imaging techniques with the current approaches of structural determination would allow a high resolution structural analysis of different α-syn structural species in test tubes, cells, and living tissues. Novel cellular and biochemical approaches are required to discover the endogenous molecules that interplay with different α-syn conformations. As the answers to these questions emerge, a clear picture of the physiological function and pathogenic behavior of α-syn will be within reach, which may further provide new molecular targets and drug candidates for therapeutic intervention against α-syn related diseases.

## Author Contributions

All authors wrote the article. All authors listed, have made substantial, direct and intellectual contribution to the work, and approved it for publication.

## Conflict of Interest Statement

The authors declare that the research was conducted in the absence of any commercial or financial relationships that could be construed as a potential conflict of interest.
